# 

**Published:** 1906-05

**Authors:** 


SUBSCRIPTION $1.00

PUBLISHED MONTHLY.

### ATLANTA JOURNAL-RECORD OF MEDICINE.

MAY 14 1906

Successor to Atlanta Medical and Surgical Journal, Established 1855, and Southern Medical Record, Established 1870.

Vol. VIII. Atlanta, Ga., May, 1906. No. 2.

